# Glutamine Cooperatively Upregulates Lipopolysaccharide-Induced Nitric Oxide Production in BV2 Microglial Cells through the ERK and Nrf-2/HO-1 Signaling Pathway

**DOI:** 10.3390/antiox9060536

**Published:** 2020-06-19

**Authors:** Rajapaksha Gedara Prasad Tharanga Jayasooriya, Ilandarage Menu Neelaka Molagoda, Matharage Gayani Dilshara, Yung Hyun Choi, Gi-Young Kim

**Affiliations:** 1 Department of Food Technology, Faculty of Technology, Rajarata University of Sri Lanka, Mihintale 50300, Sri Lanka; prasadrgtj@gmail.com; 2 Department of Marine Life Sciences, Jeju National University, Jeju 63243, Korea; neelakagm2012@gmail.com (I.M.N.M.); dilsharagm@gmail.com (M.G.D.); 3 Department of Biochemistry, College of Oriental Medicine, Dong-Eui University, Busan 47227, Korea; choiyh@deu.ac.kr

**Keywords:** glutamine, nitric oxide, inducible nitric oxide synthase, nuclear factor-erythroid 2-related factor 2, heme oxygenase-1

## Abstract

Glutamine (Gln) is a nonessential α-amino acid for protein biosynthesis. However, the mechanism through which Gln regulates NO production in microglial cells is still unclear. In this study, we investigated whether the presence or absence of Gln affects NO production in lipopolysaccharide (LPS)-stimulated BV2 microglial cells. Our data revealed that Gln depletion decreased cell viability accompanied by mild cytotoxicity, and blocked LPS-induced NO production concomitant with a significant decrease in inducible NO synthase (iNOS) expression. Additionally, Gln depletion for 24 h blocked the restoration of LPS-mediated NO production in the presence of Gln, suggesting that Gln depletion caused long-term immune deprivation. In particular, sodium-coupled amino acid transporter 1 and 2 (*SNAT1* and *SNAT2*), which are the main Gln transporters, were highly upregulated in LPS-stimulated BV2 microglial cells, in the presence of Gln accompanied by NO production. Regardless of the presence of Gln, LPS positively stimulated nuclear factor erythroid 2-related factor 2 (Nrf2) and heme oxygenase-1 (HO-1) expression, and transient *Nrf2* knockdown and HO-1 inhibition stimulated LPS-induced NO production and *iNOS* expression; however, transient *Nrf2* knockdown did not affect *SNAT1* and *SNAT2* expression, indicating that Gln transporters, *SNAT1* and *SNAT2*, were not regulated by Nrf2, which downregulated the HO-1-mediated NO production. Moreover, Gln depletion significantly reduced LPS-induced extracellular signal-regulated kinase (ERK) phosphorylation; furthermore, a specific ERK inhibitor, PD98059, and transient *ERK* knockdown attenuated LPS-stimulated NO production and *iNOS* expression, in the presence of Gln, accompanied by downregulation of *SNAT1* and *SNAT2*, suggesting that the ERK signaling pathway was related to LPS-mediated NO production via SNAT1 and SNAT2. Altogether, our data indicated that extracellular Gln is vital for NO production from microglia in inflammatory conditions.

## 1. Introduction

Microglia are the resident macrophages in the brain and spinal cord, and they act as the first line of immune defense in the central nervous system (CNS) [1]. Although transient activation of microglia contributes to the maintenance of cellular homeostasis, its chronic activation causes neurodegenerative diseases, such as Alzheimer’s disease (AD), Parkinson’s disease (PD), and multiple sclerosis [2]. Once exposed to various inflammatory stimuli, such as lipopolysaccharide (LPS), microglia immediately secrete neurotoxic molecules such as nitric oxide (NO), reactive oxygen species (ROS), and inflammatory cytokines [3]. To exert immune responses, several metabolic pathways are activated in microglia. However, whether amino acids regulate microglia activation, remains poorly understood. Recently, Kittl et al. revealed that glycine promotes the migration of microglia via sodium-coupled neutral amino acid transporters (SNATs) [4]. In addition, high levels of branched-chain amino acids, such as leucine, isoleucine, and valine, induce a peculiar polarization of microglia toward the M2 state, accompanied with a strong expression of interleukin-10 (IL-10) [5]. The above studies showed that various amino acids regulate the immune properties of microglia.

Glutamine (Gln) abundantly exists in the CNS and serves as a precursor of neurotransmitters, such as glutamate (Glu), the main excitatory neurotransmitter, and γ-amino butyric acid (GABA), the main inhibitory neurotransmitter in the mammalian cortex [6]. Once activated by LPS, microglia rapidly metabolize Gln from Glu by activating Gln synthetase (GS), triggering inflammatory and pathological responses in the CNS [7], which indicates that Gln is essential for the pathological state in the CNS. In contrary, Palmieri et al. reported that GS inhibition promoted inflammatory responses in LPS-treated microglia cells, through downregulation of Gln/Glu ratios [8]. This discrepancy was caused by the imbalance of Gln/Glu ratios and deregulation of Gln homeostasis, resulting in mitochondrial dysfunction and microglial neurotoxicity [9]. In addition, a well-known amino acid transporter, SNAT2, provides central neurons with extracellular Gln, which is consequently transformed into the neurotransmitter Glu [10]. Mackenzie et al. also revealed the importance of SNAT1 in central neurons to provide metabolic fuel and glutathione precursors [11]. Nevertheless, it remains unclear whether extracellular Gln supplementation regulates NO production as an inflammatory marker, and whether it regulates SNAT1 and SNAT2 expression.

In this study, we observed that in the presence of extracellular Gln, LPS enhanced iNOS expression and NO production in BV2 microglial cells, by activating the extracellular signal-regulated kinase (ERK) signaling pathway and inducing high expression levels of *SNAT1* and *SNAT2*. Furthermore, LPS stimulated nuclear factor erythroid 2-related factor 2 (Nrf2) and heme oxygenase-1 (HO-1) expression, regardless of the presence of extracellular Gln; however, in the presence of extracellular Gln, Nrf2 and HO-1 inhibition markedly increased LPS-mediated NO production, indicating that LPS-mediated Nrf2 and HO-1 might be involved in the downregulation of LPS-mediated NO release.

## 2. Materials and Methods

### 2.1. Chemicals and Reagents

Gln, H_2_O_2_, LPS, and 3-(4,5-dimethylthiazol-2-yl)-2,5-diphenyl-tetrazolium bromide (MTT) were purchased from Sigma-Aldrich Chemical Co. (St. Louis, MO, USA). Antibodies against iNOS, Nrf2, HO-1, ERK, phospho-ERK (p-ERK), and β-actin, and silencing RNA (siRNA) for *Nrf2* (siNrf2), *ERK* (siERK), and control siRNA (siCON) were purchased from Santa Cruz Biotechnology (Santa Cruz, CA, USA). Peroxidase-labeled goat anti-rabbit immunoglobulin was purchased from KOMA Biotechnology (Seoul, Korea). Cobalt protoporphyrin (CoPP) and zinc protoporphyrin (ZnPP) were obtained from Tocris Bioscience (Bristol, UK) and PD98059 was purchased from Calbiochem (San Diego, CA, USA). Dulbecco’s Modified Eagle’s Medium (DMEM), fetal bovine serum (FBS), and antibiotics were obtained from WelGENE Inc. (Daegu, Korea). Other chemicals were purchased from Sigma-Aldrich Chemical Co.

### 2.2. Cell Culture

BV2 microglial cells were generously gifted by Professor Il-Whan Choi (Department of Microbiology, College of Medicine, Inje University, Busan, Korea). These were cultured at 37 ℃ in 5% CO_2_ in DMEM supplemented with 5% FBS and antibiotics.

### 2.3. Cell Viability

For the analysis of cell viability, BV2 microglia cells were seeded at a density of 1 × 10^5^ cells/mL in the indicated concentrations of Gln (0–2.0 mM) for 24 h and then MTT (0.5 mg/mL) was added for 30 min. Following the media removal, dimethyl sulfoxide (DMSO) was added and gently shaken for 15 min. Absorbance of dissolved formazan was determined at 570 nm by a microplate spectrophotometer (BioTek Instruments Inc., Winooski, VT, USA). In a parallel experiment, cell images were taken under phase-contrast microscopy (MACROTECH, Goyang, Gyeonggi-do, Korea).

### 2.4. Flow Cytometry Analysis

Viability (%), dead cell (%), and total viable cell counts were measured by flow cytometry. In brief, BV2 microglial cells were seeded overnight at a density of 1 × 10^5^ cells/mL in 12 well plates, and were treated with 500 ng/mL LPS, in the presence or absence of 2 mM Gln, for 24 h. H_2_O_2_ (0.8 mM) was used as a cell death-inducing control. Then, the harvested cells were washed with ice-cold PBS and stained with Muse^®^ Count & Viability Kit (MCH100102; EMD Millipore, Billerica, MA, USA) for 5 min. Viability (%), dead cell (%), and total viable cell counts were measured by the Muse^®^ Cell Analyzer (EMD Millipore).

### 2.5. NO Assay

BV2 microglial cells (1 × 10^5^ cells/mL) were plated onto 24-well plates and incubated with the indicated concentrations of Gln for 24 h, prior to stimulation with 500 ng/mL LPS for 24 h. Cell supernatants were collected and NO production was measured. In brief, the supernatants were mixed with equal volume of Griess reagents (1% sulfanilamide in 5% phosphoric acid and 0.1% naphthylethylenediamine dihydrochloride) and then incubated at room temperature for 5 min. The absorbance was measured at 540 nm by a microplate spectrophotometer (BioTeck Instruments Inc.). Nitrite concentration was determined from a sodium nitrite standard curve.

### 2.6. Reverse Transcription Polymerase Chain Reactions (RT–PCR)

Total RNA was isolated using easy-BLUE^TM^ total RNA extraction kit (iNtRON Biotechnology, Seongnam, Gyeonggi-do, Korea), according to the manufacturer’s recommendations. Genes of interest were amplified from cDNA, using RT-PCR Premix (KOMA Biotechnology) with specific primers of *iNOS* (forward 5′-CCT CCT CCA CCC TAC CAA GT-3′ and reverse 5′-CAC CCAAAG TGC TTC AGT CA-3′; 25 cycles, annealing temperature-57 ℃), *SNAT1* (forward 5′-GAG CTC AAA GAC CGG TCA CA-3′ and reverse 5′-TGA AAA ACA GCA CAG GCA CG-3′; 35 cycles, annealing temperature-60 ℃), *SNAT2* (forward 5′-TGT ACT TGC TCG CTG CTC TC-3′ and reverse 5′-CGG AAC TCC GGA TAG GGA AA-3′; 35 cycles, annealing temperature-60 ℃), and *β-actin* (forward 5′-TGT GAT GGT GGG AAT GGG TCA G-3′ and reverse 5′-TTT GAT GTC ACG CAC GAT TTC C-3′; 24 cycles, annealing temperature-57 ℃) [12,13]. *β-Actin* was used as an internal control.

### 2.7. Western Blot Analysis

Total proteins and nuclear proteins were prepared using PRO-PREP protein extraction solution (iNtRON Biotechnology) and NE-PER nuclear and cytoplasmic extraction reagents (Pierce, Rockford, IL, USA), respectively. The proteins were separated on SDS polyacrylamide gels and electrotransferred to nitrocellulose membranes (Amersham, Arlington Heights, IL, USA). The membranes were incubated with specific antibodies and were developed using the ECL reagent kit (Amersham).

### 2.8. Electrophoretic Mobility Shift Assay (EMSA)

The preparation of nuclear protein extracts was conducted using the NE-PER nuclear and cytoplasmic extraction reagents (Pierce). The DNA-protein binding assay was performed with the nuclear protein extract. Synthetic complementary Nrf2-binding oligonucleotides (5′-TMANNRTGAYNNGCRWWWW-3′) were biotinylated using the biotin 30-end DNA labeling kit (Pierce) and annealed for 1 h at room temperature. Binding reactions were carried out for 20 min in the presence of 50 ng/mL poly (dI-dC), 0.05% Nonidet P-40, 5 mM MgCl_2_, 10 mM EDTA, and 2.5% glycerol in 1× binding buffer (Pierce), with 20 fmol biotin-end-labeled target DNA and 10 μg nuclear extract. Samples were loaded onto native 4% polyacrylamide gels for 60 min in 0.5× Tris-borate/ethylenediaminetetraacetic acid (TBE) and transferred onto a positively charged nylon membrane (HybondTM-N^+^) in 0.5× TBE at 100 V for 30 min. Transferred DNAs were cross-linked to the membrane at 120 mJ/cm^2^ and detected using horseradish peroxidase-conjugated streptavidin (LightShift™ chemiluminescent EMSA kit, Thermo Fisher Scientific, Waltham, MA, USA).

### 2.9. Transfection of siNrf2 and siERK

BV2 microglial cells were seeded on 24-well plate at a density of 1 × 10^5^ cells/mL and transfected by *Nrf2*- or *ERK*-specific siRNA for 48 h. For each transfection, 450 μL media were added to 20 nM of the siRNA duplex with transfection reagent G-Fectin (Genolution Pharmaceuticals Inc., Seoul, Korea) and the entire volume was added gently to the cells.

### 2.10. Statistical Analysis

The images were captured and visualized with Chemi-Smart 2000 (Vilber Lourmat, Cedex, France) and quantified by the Scion Imaging software (http://www.scioncorp.com). All data were derived from at least three independent experiments and expressed as mean ± standard error of the median (SEM). Significant differences between groups were determined using an unpaired one-way ANOVA test with Bonferroni correction. (* and ^#^ *p* < 0.01; *** and ^###^ *p* < 0.001).

## 3. Results

### 3.1. Gln Depletion Causes Mild Cytotoxicity in BV2 Microglial Cells

**Figure 1:**
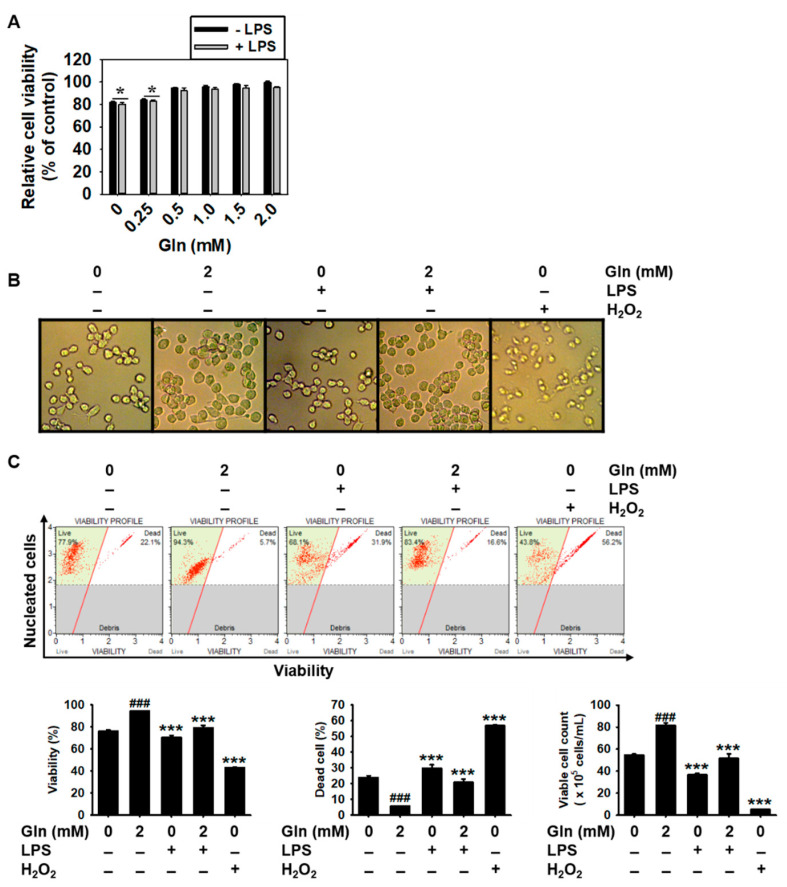
Glutamine (Gln)-free and low-Gln (below 0.25 mM) conditions decrease the viability of BV2 microglial cells. BV2 microglial cells were seeded at a density of 1 × 10^5^ cells/mL, in the presence of 2 mM Gln for 24 h, then the media were replaced with the indicated concentrations of Gln (0–2.0 mM) for 24 h, and 500 ng/mL lipopolysaccharide (LPS) or 0.8 mM H_2_O_2_ was added. (**A**) Cell viability was measured by the 3-(4,5-dimethylthiazol-2-ly)-2,5-diphenyl tetrazolium bromide (MTT) assay. The relative viability values were calculated and compared with that in the 2 mM Gln-treated cells. (**B**) Cellular morphology was examined by phase-contrast microscopy (×10). (**C**) Viability (%), dead cell (%), and viable cell count were measured by flow cytometry using Muse^®^ Cell Viability Kit. The results are the average of three independent experiments and are expressed as mean ± standard error of the median (^###^ *p* < 0.001 vs. the untreated cells; *** *p* < 0.001 and * *p* < 0.01 vs. the 2.0 mM Gln-treated cells).

To confirm the effect of extracellular Gln on the viability of BV2 microglia cells, MTT assay was performed. The absence and low concentration of Gln below 0.25 mM moderately decreased the viability of the BV2 microglial cells, regardless of the presence of LPS (Figure 1A). Furthermore, microscopic analysis showed no apoptotic characteristics such as cell shrinkage, under 2.0 mM Gln treated conditions, compared to the Gln-free conditions. To verify whether the decrease in cell viability was caused by cell death in the absence and low concentration of Gln, flow cytometric analysis was performed. The results showed that Gln free conditions resulted in 23.8 ± 1.2% cell death accompanied with 76.2 ± 1.2% cell viability and 54.6 ± 0.9 × 10^5^ cells/mL of viable cell count. Supplementation of the cells with 2.0 mM Gln restored Gln deficiency-induced cell death (94.5 ± 0.2%), viability (5.5 ± 0.2%), and viable cell count (81.4 ± 1.5 × 10^5^ cells/mL), indicating the significance of Gln to maintain normal conditions in BV2 microglial cells. Under the Gln-deficient conditions, LPS was associated with 29.8 ± 2.2% dead cell population, while the percentage was slightly decreased, up to 20.8 ± 2.2%, in the presence of 2.0 mM Gln. H_2_O_2_ at 0.8 mM significantly decreased the viability and the total cell counts of Gln. Altogether these results suggest that Gln was a pivotal amino acid to regulate normal cell survival.

### 3.2. LPS-mediated iNOS Expression and NO Production Depend on the Presence of Gln

**Figure 2:**
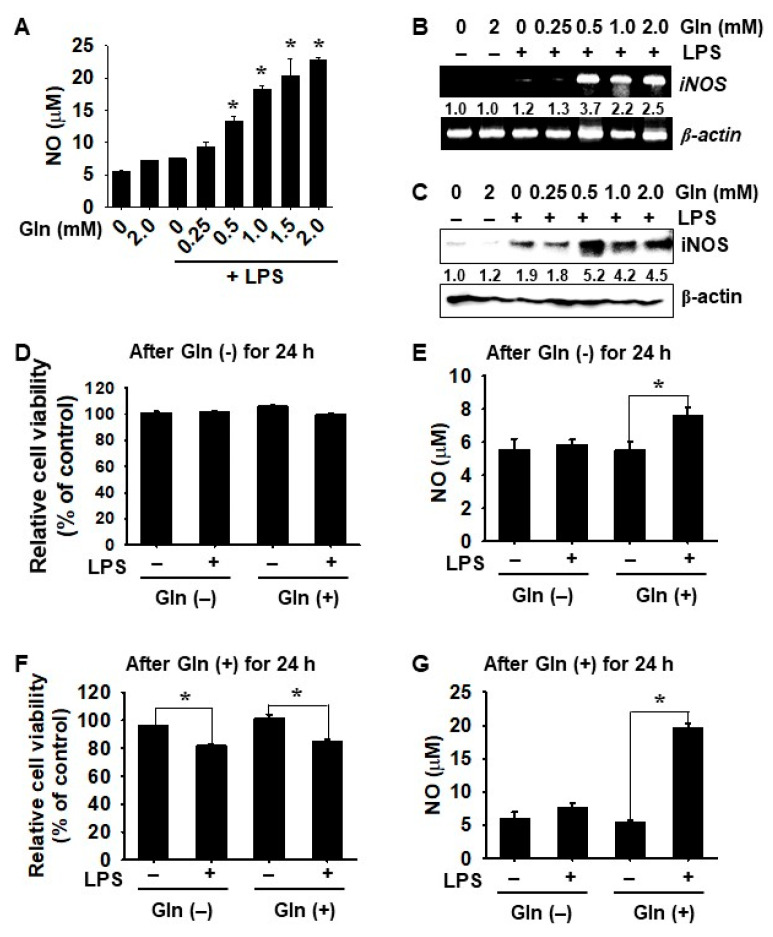
Gln-free condition downregulates lipopolysaccharide (LPS)-induced inducible nitric oxide synthase (*iNOS*) expression and nitric oxide (NO) production in BV2 microglial cells. (**A**–**C**) BV2 microglial cells (1 × 10^5^ cells/mL) were incubated with 2.0 mM Gln for 24 h, the media were then replaced with fresh media containing the indicated concentrations of Gln, 2 h before treatment with 500 ng/mL LPS. (**A**) The amount of NO were determined by the Griess reaction at 24 h and a standard curve was constructed using NaNO_2_. (**B**) In a parallel experiment, total mRNA was extracted at 6 h and RT-PCR for *iNOS* was performed. (**C**) Protein lysates were extracted at 24 h and then resolved on SDS-polyacrylamide gels. The protein was transferred to nitrocellulose membranes and probed with antibodies against iNOS. β-Actin was used as an internal control for RT–PCR and western blotting. Densitometry value of iNOS was compared to the β-actin levels in RT–PCR and western blotting; shown in the bottom. (**D**,**E**) BV2 microglial cells were cultured in Gln-free media for 24 h, and the media were replaced with Gln-free or 2.0 mM Gln-containing media, 2 h before treatment with 500 ng/mL LPS for another 24 h. (**D**) Cell viability was measured by MTT assay and (**E**) NO production was detected by the Griess reaction. (**F**,**G**) BV2 microglial cells were cultured in 2.0 mM Gln-containing media for 24 h, and the media were then replaced with Gln-free or 2.0 mM Gln-containing media for another 24 h after stimulation of 500 ng/mL LPS. (**F**) Cell viability was measured by the MTT assay and (**G**) NO production was detected by the Griess reaction. The results are the average of three independent experiments and are expressed as mean ± standard error of the median (* *p* < 0.01).

To evaluate the effects of Gln on LPS-stimulated NO production, BV2 microglial cells were stimulated with LPS for 24 h, in the presence or absence of Gln. As shown in Figure 2A, NO production was low in the presence (7.2 ± 0.1 μM) or absence (5.5 ± 0.3 μM) of 2 mM Gln, without LPS treatment. Cell stimulation with LPS resulted in a significant increase in NO production in the presence of Gln at high concentrations (13.4 ± 0.6 μM, 18.3 ± 0.7 μM, 20.4 ± 2.6 μM, and 22.8 ± 0.3 μM at 0.5, 1.0, 1.5, and 2.0 mM Gln, respectively); however, low concentrations of Gln below 0.25 mM did not affect LPS-induced NO production. In addition, RT–PCR data showed that LPS significantly increased *iNOS* expression in the presence of Gln at concentrations of over 0.5 mM after 6 h (Figure 2B). However, the absence or low concentration (0.25 mM) of Gln did not increase *iNOS* expression, even under LPS treatment. Consistent with the RT–PCR results, Western blot analysis also revealed that LPS increased *iNOS* expression in the presence of Gln over 0.5 mM at 24 h (Figure 2C). Taken together, these results indicated that Gln positively regulated *iNOS* expression and NO production in LPS-stimulated BV2 microglial cells.

Next, to further investigate the effect of extracellular Gln on cell viability and NO production, BV2 microglia cells were cultured in a Gln-free condition for 24 h and then the culture media were replaced, with or without Gln, for another 24 h. After 24 h of incubation under Gln-free conditions, no changes in cell viability (Figure 2D) and a little increase in NO production (Figure 2E) were observed, regardless of the presence of LPS. When the cells were incubated with Gln for another 24 h, cell viability and NO production were not changed, regardless of the presence of LPS, but LPS-induced NO production slightly increased (from 5.5 ± 0.7 μM to 7.6 ± 0.5 μM), which suggested that early Gln starvation for 24 h attenuated LPS-induced NO production. In a parallel experiment, BV2 microglia cells were cultured in the presence of Gln for 24 h and then the media were replaced with Gln-free or Gln-containing media, for another 24 h. Changing the cell environment to Gln-free condition slightly decreased cell viability (82.1 ± 2.4%) in the presence of LPS; however, LPS treatment did not significantly increase NO production (6.0 ± 1.0 μM). Under Gln condition for another 24 h, cell viability moderately decreased in the presence of LPS (from 101.0 ± 3.1% to 84.9 ± 1.5%) (Figure 2F), and LPS-mediated NO production significantly increased from 5.4 ± 0.4 μM to 19.6 ± 0.7 μM (Figure 2G), which indicated that Gln was required for LPS-mediated NO production. These data indicated that Gln-free conditions for 24 h irreversibly inhibited LPS-induced NO production.

### 3.3. LPS Upregulates SNAT and iNOS Expression in the Presence of Gln and Nrf2 Activation Inhibits iNOS Expression and NO Production, but not SNAT Expression

**Figure 3:**
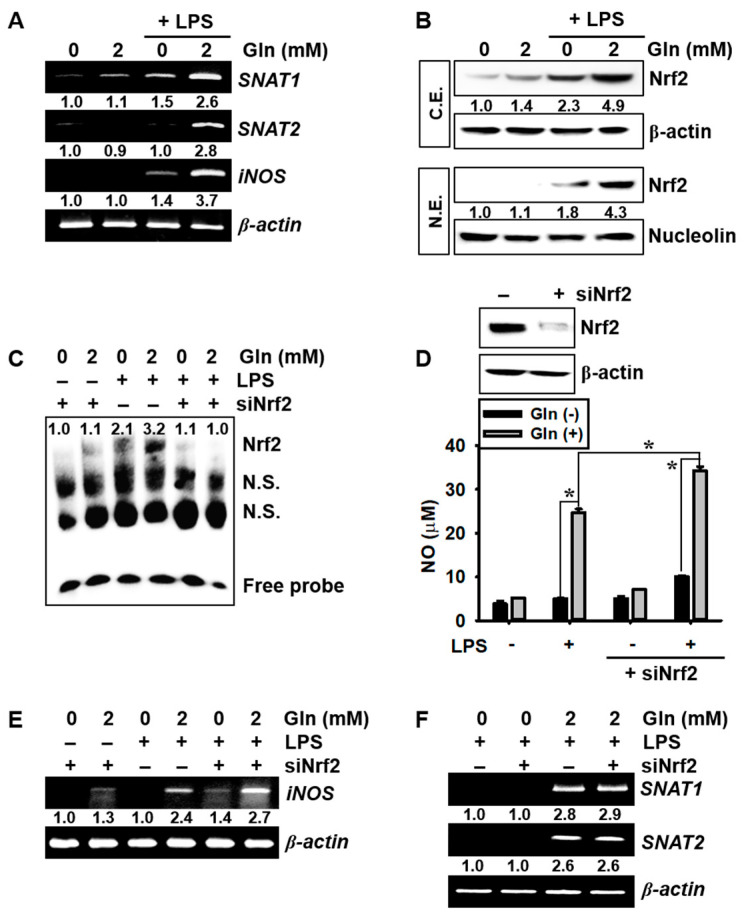
Glutamine (Gln) increases sodium-coupled amino acid transporters (*SNATs*) and inducible nitric oxide synthase (*iNOS*) expression as well as nitric oxide (NO) production, in the presence of lipopolysaccharide (LPS), but not nuclear factor erythroid 2-related factor 2 (Nrf2) expression. BV2 microglial cells (1 × 10^5^ cells/mL) were incubated with 500 ng/mL LPS in the presence or absence of Gln. (**A**) Total RNA was isolated at 6 h and RT–PCR for *SNAT1*, *SNAT2*, and *iNOS* was performed. (**B**) Nuclear and cytosolic protein was extracted at 24 h and western blotting was performed. β-Actin and nucleolin was used as the internal control for the cytosolic and nuclear fractions, respectively. Densitometry values were expressed compared to the β-actin and nucleolin levels; shown at the bottom of each figure. (**C**–**F**) BV2 microglial cells were transiently transfected with *Nrf2* siRNA (siNrf2) for 48 h in the presence of Gln and cultured with or without LPS under Gln-free condition or in the presence of Gln. (**C**) After LPS treatment for 30 min, nuclear extracts were prepared, and electrophoretic mobility shift assay was performed to assess the binding activity of Nrf2 on the anti-oxidant response element (ARE). N.S., No-specific binding of the probe. (**D**) After transfection of siNrf2 for 48 h, the level of Nrf2 expression was detected by western blotting (**top**). The amount of NO were determined by the Griess reaction, and a standard curve was constructed using NaNO_2_ (**bottom**). (**E**) The expression of *iNOS* and (**F**) *SNAT1/2* was detected at 6 h by RT–PCR. β-Actin was used as an internal control for RT–PCR. Densitometry values were expressed compared to the β-actin levels and displayed at the bottom of each figure. The results are the average of three independent experiments and are expressed as mean ± standard error of the median (* *p* < 0.01).

To clarify the role of Gln in LPS-induced *SNAT* expression and NO production, BV2 microglial cells were stimulated with LPS for 6 h in the presence or absence of Gln. Our data revealed that LPS significantly increased both *SNAT1* and *SNAT2* expression and induced high *iNOS* expression in the presence of Gln. In contrast, the Gln-free condition slightly increased *SNAT1*, *SNAT2*, and *iNOS* expression even in the presence of LPS (Figure 3A). We also investigated whether Nrf2 regulates LPS-induced *SNAT* expression and NO production. LPS promoted the upregulation of total Nrf2 levels, concomitant with Nrf2 nuclear translocation (Figure 3B) and specific DNA-binding activity (Figure 3C), regardless of the presence of Gln, which indicated that Nrf2 expression was dependent on the presence of LPS, not Gln. In addition, transient knockdown of *Nrf2* using siRNA significantly increased LPS-mediated NO production to 37.1 ± 1.3 μM, in the presence of Gln, compared with that in the LPS-treated group without siNrf2 transfection (26.2 ± 1.1 μM) (Figure 3D). Moreover, siNrf2 transfection markedly increased *iNOS* expression in the presence of Gln, compared with that in the LPS-treated group without siNrf2 transfection (Figure 3E), indicating that Nrf2 was activated by LPS, regardless of the extracellular existence of Gln, which inhibited *iNOS* expression and NO production. However, Gln-induced *SNAT1* and *SNAT2* expression under the LPS-treated condition was unaffected by the siNrf2 transfection. (Figure 3F), suggesting that the expression of *SNAT1* and *SNAT2* was not dependent on Nrf2 activity. These data indicated that extracellular Gln promoted LPS-mediated *SNAT1*, *SNAT2,* and *iNOS* expression, as well as Nrf2 activation. In addition, Nrf2 activation was found to be related to the presence of LPS, but not Gln.

### 3.4. LPS Promotes HO-1 Expression Regardless of the Presence of Gln, which Decreases NO Production

**Figure 4:**
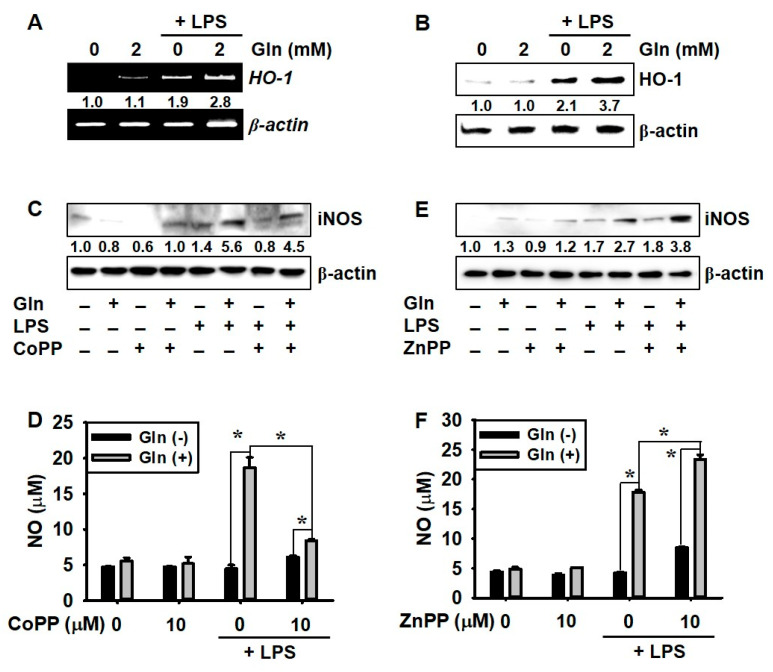
Glutamine (Gln) increases lipopolysaccharide (LPS)-mediated heme oxygenase-1 (HO-1) expression and nitric oxide (NO) production. BV2 microglial cells (1 × 10^5^ cells/mL) were incubated in the presence or absence of Gln 2 h before being treated with 500 ng/mL LPS. (**A**) Total mRNA was isolated 6 h after LPS treatment, and RT–PCR for *HO-1* was performed. (**B**) In a parallel experiment, protein lysates were prepared at 24 h, and equal amounts of the lysates were resolved on SDS-polyacrylamide gels, transferred to nitrocellulose membranes, and probed with a specific antibody against HO-1. (**C**,**D**) BV2 microglia cells were pretreated with 10 μM cobalt protoporphyrin (CoPP) for 1 h and then, incubated with LPS for 24 h, in the presence and absence of 2.0 mM Gln. (**C**) Western blotting was performed to check *iNOS* expression. (**D**) NO production was measured by Griess reaction. (**E**,**F**) BV2 microglia cells were pretreated with 10 μM zinc protoporphyrin (ZnPP) for 1 h, and then incubated with LPS for 24 h in the presence or absence of 2.0 mM Gln. (**E**) Western blotting was performed to check *iNOS* expression. (**F**) NO production was measured by the Griess reaction. β-Actin was used as the internal control for RT–PCR and western blotting. Densitometry values were expressed, as compared to the β-actin levels; these are displayed on each figure. The results are the average of three independent experiments and are expressed as the mean ± standard error of the median (* *p* < 0.01).

As Nrf2-dependent adaptive response is a pivotal defense mechanism against nitrosative stress through HO-1 activation [14], we examined whether HO-1 downregulates NO production in the presence of Gln. Gln alone (in the absence of LPS) did not remarkably upregulate *HO-1* expression, as compared to the Gln-free condition; however, LPS-mediated *HO-1* expression was significantly higher in the presence of Gln and, in the absence of Gln, LPS moderately increased *iNOS* expression (Figure 4A). Consistent with the data on *HO-1* expression, almost no HO-1 protein was expressed in LPS-unstimulated conditions in BV2 microglial cells, regardless of the presence of Gln. However, in the presence of Gln, LPS-mediated HO-1 expression further increased, even in the absence of Gln (Figure 4B). Moreover, an HO-1 inducer, CoPP, significantly decreased LPS-induced *iNOS* expression (Figure 4C) and NO production from 18.6 ± 1.5 μM to 8.4 ± 0.2 μM, in the presence of Gln (Figure 4D). In contrast, pretreatment with an HO-1 inhibitor, ZnPP, further increased LPS-mediated *iNOS* expression (Figure 4E) and NO production from 17.6 ± 1.2 μM to 24.1 ± 2.3 μM in BV2 microglia cells (Figure 4E). Taken together, these results indicated that LPS-mediated HO-1 expression downregulated NO production, but LPS-stimulated NO production overcame the HO-1-mediated inhibition.

### 3.5. ERK Phosphorylation Upregulates LPS-Induced NO Production in the Presence of Gln 

**Figure 5:**
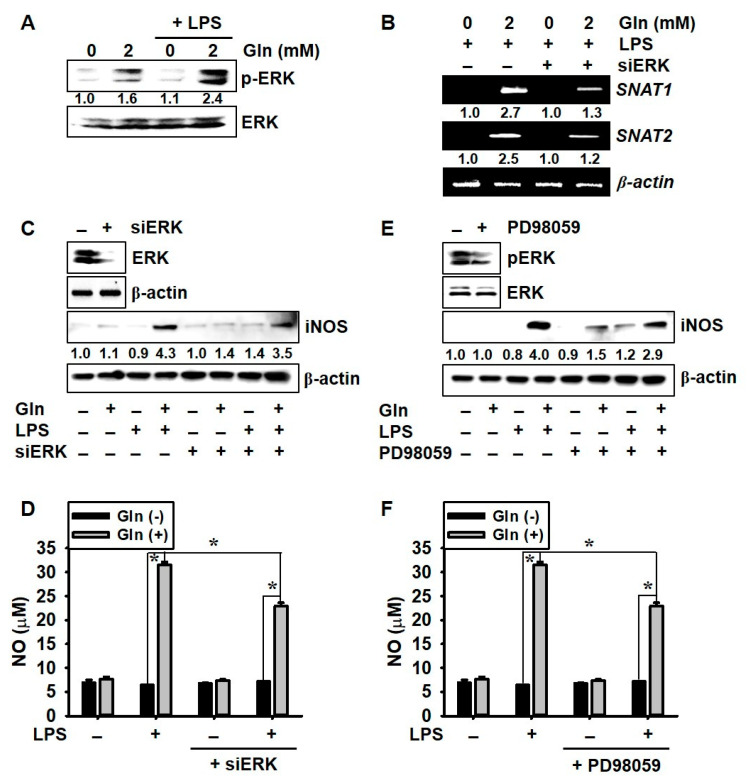
Glutamine (Gln) promotes extracellular signal-regulated kinase (ERK) phosphorylation and nitric oxide (NO) production as well as the expression of sodium-coupled amino acid transporter 1 (*SNAT1*) and *SNAT2*, under lipopolysaccharide (LPS) stimulation. BV2 microglial cells were incubated with 2.0 mM Gln, 2 h before treatment with 500 ng/mL LPS. (**A**) After 24-h incubation with LPS, protein lysates were resolved on the SDS-polyacrylamide gels, transferred to nitrocellulose membranes, and probed with specific antibodies against p-ERK and ERK. Total ERK was used as an internal control. Densitometry values were expressed, as compared to the total ERK levels; displayed at the bottom of the figure. (**B**) Total mRNA was isolated at 6 h and RT–PCR for *SNAT1* and *SNAT2* was performed. β-Actin was used as an internal control for RT–PCR. Densitometry values were expressed compared to the β-actin levels; displayed at the bottom of the figure. (**C**,**D**) The cells were transiently transfected with *ERK*-targeting siRNA (siERK) for 48 h. (**C**) Western blotting was performed to check ERK phosphorylation (top) and *iNOS* expression (bottom). (**D**) NO production was measured by the Griess reagent assay. (**E**,**F**) The cells were also preincubated with an ERK inhibitor, PD98059 (20 μM), for 2 h and were treated with LPS for 24 h. (**E**) Western blotting was performed to check ERK phosphorylation (top) and *iNOS* expression (bottom). (**F**) NO production was measured by Griess reaction. β-Actin was used as an internal control for *iNOS* protein expression. Densitometry values were expressed as compared to the β-actin levels; displayed at the bottom of the figure. The results are the average of three independent experiments and are expressed as the mean ± standard error of the median (* *p* < 0.01).

Considering that the ERK signaling pathway increases *SNAT1* and *SNAT2* expression and NO production [15], we examined the functional role of ERK in the presence of Gln. In the Gln-free condition, the cells showed a very low level of ERK phosphorylation (Figure 5A). On the contrary, ERK phosphorylation markedly increased in the presence of Gln, and LPS further upregulated ERK phosphorylation. In addition, the transient knockdown of *ERK* moderately downregulated LPS-induced *SNAT1* and *SNAT2* expression (Figure 5B), which indicated that ERK partially activated *SNAT1* and *SNAT2* expression in the presence of Gln. We also confirmed whether the ERK signaling pathway regulates LPS-induced NO production. Both silencing of *ERK* and an ERK inhibitor, PD98059, decreased LPS-mediated *iNOS* expression (Figure 5C,E) and NO production (Figure 5D,F), in the presence of Gln, which indicated that in the presence of Gln, ERK partially upregulated NO production in the LPS-treated BV2 microglia cells. These data indicated that ERK upregulated NO production cooperatively with Gln and induced high expression of *SNAT1* and *SNAT2*.

## 4. Discussion

Microglia and astrocytes are the major immune-regulating cells in the CNS [16]. Excessive NO production from microglia is linked to the major pathogenesis of several neurodegenerative diseases, such as AD and PD, resulting in neuronal cell death [17,18]. A previous study reported that, upon stimulation with LPS, the consumption of amino acids as cellular energy sources and nutrients is significantly increased in macrophages, resulting in a dramatic increase in NO production [19]. In the current study, we elaborated that Gln plays a crucial role in LPS-stimulated NO production, through the ERK signaling pathway. In addition, LPS simultaneously activated the Nrf2/HO-1 pathway, regardless of the presence of Gln, and the Nrf2/HO-1 pathway subsequently inhibited NO production, but not the *SNAT1* and *SNAT2* expression.

Gln is a ubiquitous amino acid in the CNS and its main role is to supply neurotransmitters, such as Glu and GABA, through GS activity [6]. Recently, Nakajima et al. suggested that, once activated with LPS in the presence of Glu, microglia rapidly uptake Glu and significantly metabolizes Gln via GS activation, indicating that the metabolism of Gln from Glu (an increase of intracellular Gln) activates microglia, causing inflammatory and pathological responses in the CNS [7]. To confirm the functional role of extracellular Gln, in the current study, we investigated NO production in the presence or absence of extracellular Gln. Our data showed that LPS-treated BV2 microglial cells did not show *iNOS* expression and NO production under the Gln-free condition; however, the addition of Gln significantly increased the LPS-mediated NO production, implying that Gln was an essential molecule for LPS-mediated NO production. In this regard, Gln supplementation might aggravate neurodegenerative diseases through activation of microglia, along with high levels of NO. On the contrary, Palmieri et al. found that microglia-specific GS inhibition (a decrease in intracellular Gln) increased inflammatory mediators, such as NO and ROS, as well as reduced intracellular Gln/Glu ratio in autoimmune encephalomyelitis models, which indicated that a decrease in intracellular Gln exacerbated neuroinflammation [8]. Another study revealed that Gln intake significantly reduced neurodegeneration in AD models, and reduced tau phosphorylation, DNA damage, and synaptic protein loss [20]. Recently, preclinical supplementation of Gln also showed potential as a therapeutic strategy for the treatment of childhood genetic neurodegenerative disease, ataxia-telangiectasia [21]. To solve the discrepancy of the role of Gln on inflammation, how GS and glutaminase are peculiarly activated at the different concentrations of extracellular and intracellular Gln should be investigated. In conclusion, intracellular Gln could drive both Glu-ergic (inflammatory) or GABA-ergic (anti-inflammatory) transmission, depending on the regulation of intracellular Gln metabolism, including its transportation from an extracellular location, which is associated with different pathological conditions of the CNS [22]. In addition, Glu-ergic or GABA-ergic transmission contributes to the degree of glucose oxidation [23], which indicates that the role of Gln in vivo must be investigated with comprehensive nutritional considerations.

SNATs are the most well-known Gln transporters, and they unidirectionally carry a sodium ion with a neutral amino acid such as Gln from the extracellular environment to the intracellular compartment, leading to the Glu-ergic or GABA-ergic cycle [24]. Jin et al. reported that the Rett syndrome is an autism spectrum disorder caused by loss-of-functions in the gene encoding MeCP2, which is a repressor of *SNAT1* expression in microglia [9]. Moreover, dysregulation of *SNAT1* causes substantial disruption in microglial Gln homeostasis and mitochondrial function, and it also disturbs the Glu-ergic or GABA-ergic cycle [23]. Yamada et al. found that *SNAT1* protects neuronal cell death against ischemic brain damage via the mTOR-autophagy system [25]. The above data support that *SNAT1*-mediated Gln uptake inhibits neuroinflammation by activating GABA-ergic transmission [26]. Furthermore, *SNAT2* is well-known to be primarily expressed in Glu-ergic neurons, which trigger Glu conversion from Gln [27]; however, Grewal et al. revealed that SNAT2 regulated Gln transporter, but not for Glu-ergic transmission [10]. Accumulating evidence showed that *SNAT1* and *SNAT2* are crucial Gln transporters that regulate immune responses and homeostasis in central neurons, but their roles are contradictory. In addition, the underlying mechanism of Gln regulation of microglia activation via *SNATs* is still unclear. In the current study, extracellular Gln stimulated both *SNAT1* and *SNAT2* expression in LPS-stimulated BV2 microglial cells, as well as Nrf2-mediated HO-1 expression. Lister et al. reported that *SNAT1* was remarkably upregulated in *Nrf2*-knockout-metabolic acidosis mice, but not *SNAT2* [28]. Nevertheless, *SNAT*-mediated NO production has not been investigated. Additionally, the activation of the Nrf2 pathway inhibits the progression of inflammation by stimulating HO-1 release from Keap1 [29]. Free HO-1 is responsible for the oxidative cleavage of heme groups, leading to the generation of biliverdin, carbon monoxide, and release of ferrous iron [30], which plays a role in immunoinflammatory diseases and might represent a therapeutic target [31]. Indeed, defective expression of HO-1 is associated with relapses of multiple sclerosis and treatment of rodent models of MS by HO-1 inducers, CO inhalation, or systemically-administered CO donors, which improve the clinical and histological course of the disease with profound effects on immune responses [32,33]. In this study, Nrf2 and HO-1 inhibition significantly increased *iNOS* expression and NO production, but did not affect both *SNAT1* and *SNAT2* expression, which indicated that Nrf2-mediated HO-1 suppressed LPS-mediated *iNOS* expression and NO production, regardless of the expression of *SNAT1* and *SNAT2*. Our data imply that extracellular Gln increases *SNAT* expression, which consequently stimulates LPS-mediated *iNOS* expression and NO production, concomitant with an increase of Nrf2 and HO-1 expression; however, no relation was found between increased *Nrf2* and *SNAT* expression. Additionally, LPS-induced Nrf2 and HO-1 increase acts to inhibit *iNOS* expression and NO production, which cannot be suppressed in the presence of Gln. Therefore, further studies are needed to evaluate whether Nrf2 regulate *SNAT* expression through different manners, in many pathological disorders. Moreover, our data showed that the presence of Gln partially increased ERK-mediated *SNAT1* and *SNAT2* expression in LPS-treated BV2 microglia cells, which was not activated under Gln-free conditions. We also elaborated that LPS-induced NO production was stimulated through SNAT-mediated Gln transportation. Finally, although Chen and Herrup showed that Gln intake reduced neurodegeneration in AD models [20], we need to carefully access Gln intake to cure neuroinflammation as excessive Gln can stimulate an amount of NO production, causing exacerbation of inflammation.

**Figure 6:**
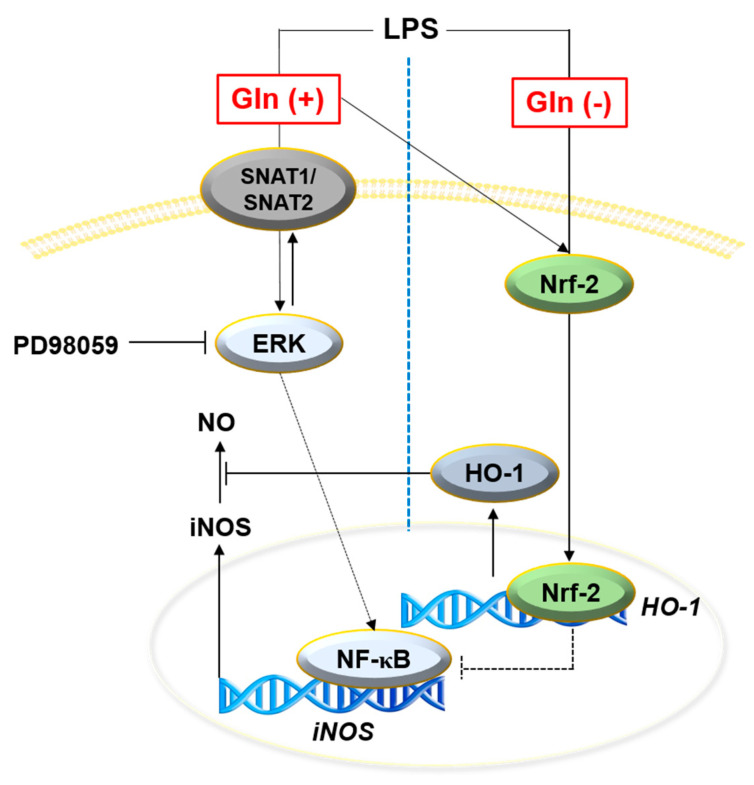
Glutamine (Gln) cooperatively regulates lipopolysaccharide (LPS)-mediated nitric oxide (NO) production. In the presence of Gln, LPS activates the expression of *SNAT1* and *SNAT2* through ERK phosphorylation, which increases Gln uptake and NO production. In addition, the Nrf2/HO-1 pathway decreases LPS-mediated NO production, but the pathway cannot inhibit LPS-mediated NO overproduction. On the contrary, in a Gln-free condition, *SNAT1* and *SNAT2* expression is low, and ERK phosphorylation is minimum, even in the presence of LPS; however, Nrf2 and HO-1 are still expressed, inhibiting LPS-mediated NO production. These data indicate that Gln is an essential molecule for LPS-mediated NO production.

In summary, extracellular Gln increased *iNOS* expression and NO production in LPS-treated BV2 microglia cells, by activating the ERK signaling pathway (Figure 6). Nrf2-mediated *HO-1* was upregulated in the presence of LPS, regardless of the presence of extracellular Gln, and tried to downregulate *iNOS* and NO production. In this study, extracellular Gln boosted microglia, which promoted NO production, causing neuroinflammation and neurodegenerative diseases. Nevertheless, whether Gln-mediated NO production leads to detrimental effects in CNS requires further studies, since NO is known to be an important messenger in cellular communication, depending on its physiological concentration [34,35]. Finally, whether native microglia in the presence of Gln also regulate *iNOS* expression and NO production in the same way as with BV2 microglia cells, needs to be investigated, since the compatibility of BV2 microglial cells with native microglia remains an issue.
